# Investigation of Prolific Sheep from UK and Ireland for Evidence on Origin of the Mutations in *BMP15* (*Fec*X*^G^*, *Fec*X*^B^*) and *GDF9* (*Fec*G*^H^*) in Belclare and Cambridge Sheep

**DOI:** 10.1371/journal.pone.0053172

**Published:** 2013-01-02

**Authors:** Michael P. Mullen, James P. Hanrahan, Dawn J. Howard, Richard Powell

**Affiliations:** 1 Department of Microbiology, National University of Ireland, Galway, Ireland; 2 Teagasc, Animal and Grassland Research and Innovation Centre, Athenry, Co. Galway, Ireland; Institut National de la Recherche Agronomique-CNRS UMR6175, France

## Abstract

This paper concerns the likely origin of three mutations with large effects on ovulation rate identified in the Belclare and Cambridge sheep breeds; two in the *BMP15* gene (*Fec*X*^G^* and *Fec*X*^B^*) and the third (*Fec*G*^H^*) in *GDF9*. All three mutations segregate in Belclare sheep while one, *Fec*X*^B^*, has not been found in the Cambridge. Both Belclare and Cambridge breeds are relatively recently developed composites that have common ancestry through the use of genetic material from the Finnish Landrace and Lleyn breeds. The development of both composites also involved major contributions from exceptionally prolific ewes screened from flocks in Ireland (Belclare) and Britain (Cambridge) during the 1960s. The objective of the current study was to establish the likely origin of the mutations (*Fec*X*^G^*, *Fec*X*^B^* and *Fec*G*^H^*) through analysis of DNA from Finnish Landrace and Lleyn sheep, and Galway and Texel breeds which contributed to the development of the Belclare breed. Ewes with exceptionally high prolificacy (hyper-prolific ewes) in current flocks on Irish farms were identified to simulate the screening of ewes from Irish flocks in the 1960s. DNA was obtained from: prolific ewes in extant flocks of Lleyn sheep (*n* = 44) on the Lleyn peninsula in Wales; hyper-prolific ewes (*n* = 41); prolific Galway (*n* = 41) ewes; Finnish Landrace (*n* = 124) and Texel (*n* = 19) ewes. The *Fec*X*^G^* mutation was identified in Lleyn but not in Finnish Landrace, Galway or Texel sheep; *Fec*X*^B^* was only found among the hyper-prolific ewes. The *Fec*G*^H^* mutation was identified in the sample of Lleyn sheep. It was concluded from these findings that the Lleyn breed was the most likely source of the *Fec*X*^G^* and *Fec*G*^H^* mutations in Belclare and Cambridge sheep and that the *Fec*X*^B^* mutation came from the High Fertility line that was developed using prolific ewes selected from commercial flocks in Ireland in the 1960′s and subsequently used in the genesis of the Belclare.

## Introduction

Studies of variation in ovulation rate and litter size in populations of prolific sheep led to the conclusion that gene(s) with a large effect on prolificacy segregate in several breeds, including the Booroola [1], [2], Romney [3], Cambridge [4], Belclare [5], Icelandic [6], Javanese [7], Olkuska [8], Lacaune [9] and Woodlands sheep [10]. Subsequent studies led to the identification of the genes responsible in most of these cases: the *FecB^B^* mutation in *BMPR1B* (bone morphogenetic protein receptor type 1B) in Booroola Merino [11] and Javanese [12] sheep; mutations in *BMP15* (bone morphogenetic protein 15) in Romney (*Fec*X*^I^*, *Fec*X*^H^)* [13], Cambridge and Belclare (*Fec*X*^G^*, *Fec*X*^B^*) [14], Lacaune (*Fec*X*^L^)* [15], and Rasa Aragonesa sheep *(Fec*X*^R^*) [16], [17]; and mutations in *GDF9* (growth differentiation factor 9) in Belclare, Cambridge (*Fec*G*^H^)* [14], Icelandic [*Fec*G*^T^*] [18] and Santa Inês sheep (*Fec*G*^E^*) [19].

Exceptional prolificacy in the Cambridge and Belclare breeds has been shown to be due to mutations in two oocyte-derived growth factor genes: the X-linked *BMP15* (*Fec*X*^G^* and *Fec*X*^B^* mutations), and *GDF9* (*Fec*G*^H^* mutation) on chromosome 5 [14]. Heterozygous carriers of the mutations in *BMP15* exhibit an increase in ovulation rate (+0.9; [14]) similar to that seen in Inverdale and Hanna sheep [20] while sheep heterozygous for *Fec*G*^H^* exhibit a greater increase in ovulation rate (+1.8; [14]). The ovulation rate observed in double heterozygotes (e.g., *Fec*X*^G^/Fec*X*^+^; Fec*G*^H^/Fec*G*^+^)* reflects an essentially additive effect of these mutations on this trait [14]. However, homozygous carriers of *Fec*G*^H^* or of either of the mutations in BMP15 and ewes with the genotype *Fec*X*^G^*/*Fec*X*^B^,* are sterile due to arrested follicle development [14].

The Belclare and Cambridge breeds are relatively recently developed composites formed with high prolificacy as the primary objective. While the procedures employed differed, ewes selected, from commercial flocks, because of their exceptionally high prolificacy contributed to the formation of both breeds. Cambridge sheep were developed from a set of 54 foundation ewes, mostly purebred, with exceptionally high prolificacy screened from flocks in Britain during the 1960s; these foundation ewes included three representatives of the Lleyn breed. The foundation ewes were joined initially with Finnish Landrace rams and subsequent matings involved backcrossing the resulting ½ Finn rams onto foundation ewes [21]. The Belclare breed was developed initially by combining three genetic stocks: a High Fertility line, an interbred Finn × Galway line and a flock of Lleyn sheep [5]. The High Fertility line was derived from ewes with exceptional prolificacy that were screened from flocks throughout Ireland in the early 1960s [22]. The Lleyn sheep used were from a flock developed from ewes and rams imported into Ireland in 1976 [23]; the ewes imported had the highest litter size records in recorded flocks in north Wales in the mid 1970s and the rams were born to ewes of equivalent prolificacy. There was no selection of the either the Galway or Finnish Landrace components in the Finn × Galway line. Subsequent development of the Belclare involved the introduction of genetic material from the Texel breed through ¾Texel males from planned matings with Finnish Landrace ewes from a line selected for high ovulation rate [24], [25]. Thus, there were three common elements in the formation of the Belclare and Cambridge: intense screening from commercial flocks, genetic material from the Finnish Landrace breed and purebred Lleyn sheep with a high prolificacy record. The genetic links between Cambridge and Belclare are essentially through the Finnish Landrace and the Lleyn breeds although there were a few crossbred foundation ewes in the Cambridge with Suffolk and Cheviot ancestry [21] and these breeds also contributed to the High Fertility line [26].

Since the Lleyn and Finnish Landrace were the most direct common ancestors of Belclare and Cambridge the first objective was to examine the hypothesis that the Lleyn was the likely source of the mutations that were common to these breeds as the available evidence indicated that genes with a major effect on ovulation rate do not contribute to the high prolificacy of Finnish Landrace sheep [24], [27]. A second objective was to seek evidence for the presence of the *BMP15* mutation unique to the Belclare breed among prolific ewes on farms in Ireland or in the other breeds that were major contributors to the Belclare. Brief summaries on preliminary results from this study have been published [28], [29].

## Materials and Methods

### Animals

#### Ethics statement

Animal handling and blood collection was conducted under license issued in accordance with Irish and European Union legislation (Cruelty to Animals Act, 1876, and European Community Directive, 86/609/EC). All animals were managed in accordance with the guidelines for the accommodation and care of animals under Article 5 of the Directive.

#### Finnish landrace sheep

The Finnish Landrace breed was represented by ewes from two lines (High and Low) developed by divergent selection on ovulation rate [24] plus ewes from an unselected control line. The animals involved were born in 1994 and 1995 and represented 37 High, 38 Low and 49 Control females.

#### Hyper-prolific ewes

**Figure 1:**
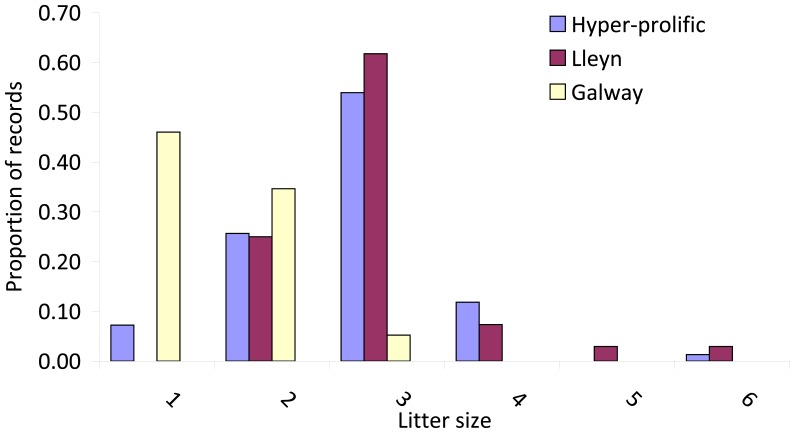
Litter size records for Hyper-prolific (n = 41), Lleyn (n = 32) and Galway (n = 41) ewes (representing 152, 68 and 113 litter size records, respectively) selected for the study. In the case of the Lleyn a litter size of 3 includes 5 cases with “at least 3″ offspring per lambing.

In an attempt to replicate the original screening of prolific ewes from Irish flocks, ewes that met the criteria used in the 1960s (viz., having produced three sets of triplets or better [22]) were sought through advertisements in the farming press. This elicited a set of 65 ewes, representing 28 flocks throughout the Republic of Ireland. Information on the litter size record and available knowledge regarding the ancestry of each ewe was recorded during a follow-up visit to obtain a sample of blood. Twenty four of these cases, representing seven flocks, were declared to have Belclare ancestry and were excluded from the study, leaving a set of 41 individuals, referred to as hyper-prolific (HP) ewes hereinafter. Available information on the litter size record of these HP ewes is summarized in Fig. 1.

#### Texel and galway sheep

Blood samples were collected from 19 Texel ewes in the flock maintained at Teagasc, Athenry, Co. Galway, Ireland and from 41 pedigree Galway ewes with the highest litter size performance based on information from the performance recording programme for this breed (see Fig. 1) [30].

### Lleyn Sheep

#### Selected ewes from specific flocks

Blood samples were obtained from ewes in six different flocks of Lleyn sheep in the Lleyn peninsula in Wales where flocks that contributed the Lleyn sheep used in the formation of the Belclare were located. The flocks involved included two of the flocks that contributed to the original importation into Ireland in the 1970s. Animal selection was primarily aimed at ewes with a history of triplet births, although individual written records were not available in all cases, and the final set of 44 individuals sampled included female progeny of ewes with exceptional prolificacy. Available litter size records of the selected ewes are summarized in Fig. 1.

#### Population survey

Arising from the detection of mutations in *BMP15* (*Fec*X*^G^)* and *GDF9* (*Fec*G*^H^*) among the selected sample of Lleyn ewes a population survey was undertaken to ascertain the frequency of these mutations in the Lleyn breed. The opportunity was also taken to screen for all mutations in these genes that were known at the time of the survey as well as for the Booroola mutation. Blood samples were obtained from the population of 18-month old rams presented at registration inspections throughout Britain and Ireland in summer 2009. A total of 333 rams were genotyped and these represented 140 flocks.

### Blood Sampling

Samples from Finnish Landrace, Texel and the selected Lleyn ewes were collected in 10 ml EDTA vacutainers while samples were collected on FTA® paper (Whatmann Bioscience, Cambridge, UK) in the case of the Galway and HP ewes. In the case of the survey of Lleyn population blood samples were collected on FTA® paper using ‘do-it-yourself’ DNA sampling kits (Innovis Ltd., Aberstwyth, UK).

### Genotyping

With the exception of the samples for the survey of the Lleyn population, animals were genotyped using PCR-amplified DNA and forced restriction fragment length polymorphism (RFLP) assays (PCR-RFLP), as described previously, using genomic DNA isolated from white blood cells in whole blood or from DNA on FTA® paper [14], [31]. Briefly, PCR amplified and digested DNA fragments were separated on 4% agarose gels and visualized with ethidium bromide staining. The gels were scored for the presence or absence of the mutations. Homozygous, heterozygous and negative controls were included in each PCR-RFLP assay.

DNA samples from the Lleyn population survey were analyzed, using the Sequenom MassArray® iPLEX Gold assay (Eurofins-Medigenomix, Ebersberg, Germany), for seven mutations known, at the time of genotyping, to be associated with ovarian function in sheep. These included five mutations in *BMP15* (*Fec*X*^G^*, *Fec*X*^B^*, *Fec*X*^I^*, *Fec*X*^H^*, *Fec*X*^L^),* the Booroola gene (*Fec*B*^B^*) and *Fec*G*^H^* in *GDF9*.

### DNA Sequencing

To validate the PCR-RFLP results the DNA from 17 selected carriers, representing *Fec*X*^G^*, *Fec*X*^B^* or *Fec*G*^H^*, were sequenced for the second exon of both *BMP15* and *GDF9* as previously described [14] (EuroFins-Medigenomix).

In order to explore evidence related to the likely source of the mutations DNA from a set 40 individuals, chosen from the available Cambridge, Belclare, Lleyn and HP animals, representing heterozygous and homozygous carriers of *Fec*X*^G^*, *Fec*X*^B^* or *Fec*G*^H^* along with wild type individuals, was sequenced. The complete coding regions of both *GDF9* and *BMP15* were amplified using the PCR conditions, including primers, described by Hanrahan et al. [14] and assessed using published sheep sequences (sheep genomic *BMP15* exon 1, *AF236078*; sheep genomic *BMP15* exon 2, *AF236079*; sheep genomic *GDF9* exon 1 and 2, *AF078545*).

## Results

### Genotyping

The PCR-RFLP genotyping of the original 44 Lleyn sheep led to the identification of 12 heterozygous carriers of *Fec*X*^G^* and a single heterozygous carrier of *Fec*G*^H^*. No carriers of *Fec*X*^B^* were detected in the Lleyn sheep sampled (Table 1). The set of 41 HP ewes included five ewes that were heterozygous for *Fec*X*^B^* and one *Fec*G*^H^* heterozygote (Table 1); no carriers of *Fec*X*^G^* were detected. None of the 124 Finnish Landrace sheep were carriers of *Fec*X*^G^*, *Fec*X*^B^* or *Fec*G*^H^*. Neither were any of these mutations detected among the Texel or Galway sheep tested. Information on the location and breed type of the individual carriers detected among the set of HP ewes is presented in Table 2.

**Table 1:** Incidence of carriers of mutations in *BMP15* and *GDF9* among selected Lleyn and Hyper-prolific (HP) ewes from commercial flocks.

Group	Number of sheep tested	Genotype		
		*Fec*X*^G^/+*	*Fec*X*^B^/+*	*Fec*G*^H^/+*
Lleyn	44	12	0	1
HP	41	0	5	1

**Table 2:** Details on heterozygous carriers among Hyper-prolific ewes in commercial flocks.

Heterozygous carrier of	Individual identifier†	Location of flock	Breed description	Litter size record
*Fec*X*^B^*	E2170616∶10	Fanad, Co. Donegal	Milford x Texel	3,3,3
*Fec*X*^B^*	H2351074∶5	Caherciveen, Co. Kerry	Cheviot x Texel	2,4
*Fec*X*^B^*	A1150505∶151	Bunclody, Co. Wexford	Suffolk-x	2,3,3,4,4
*Fec*X*^B^*	X1930375∶276‡	Collinstown, Co. Westmeath	Suffolk-x	1,4
*Fec*X*^B^*	X1930375∶277‡	Collinstown, Co. Westmeath	Suffolk-x	2,4
*Fec*G*^H^*	P1371043∶6	Tuam, Co. Galway	Suffolk-x	3,3,3,3

† National Sheep Identifier.

‡ Full sisters from the same flock.

#### Survey of lleyn population

The set of 333 Lleyn rams yielded six carriers of *FecX^G^*, nine heterozygous carriers of *FecG^H^* and one ram that was homozygous *Fec*G*^H^/Fec*G*^H^*. None of the other known (at the time of the survey) mutations with a major effect on ovulation rate (*Fec*X*^B^*, Inverdale (*Fec*X*^I^*), Hanna (*Fec*X*^H^*), Lacaune (*Fec*X*^L^*) and Booroola (*Fec*B*^B^*)) was detected in the Lleyn sheep tested. The estimated frequencies for the *FecX^G^* and *FecG^H^* mutations in the Lleyn population are presented in Table 3. Carriers were detected in 10% of the 140 flocks that contributed to the survey, which yielded a 95% confidence interval estimate of 6.0 to 15.9% for the incidence of Lleyn flocks with at least one of these mutations.

**Table 3:** Estimates of gene frequency for mutations in *GDF9* and *BMP15* in a sample of 333 Lleyn rams representing 140 flocks in Britain and Ireland in 2009.

Gene	Mutation	Frequency (%) of mutation	
		Estimate	95% Confidence interval†
*GDF9*	*Fec*G*^H^*	1.7	0.7 to 2.8
		(3.1)‡	(1.5 to 5.6)
*BMP15*	*Fec*X*^G^*	2.0	0.6 to 3.7

† Based on variation among 10 000 bootstrap samples.

‡ Incidence of carrier animals.

### DNA Sequencing

In all 17 cases examined the sequencing results confirmed the genotypes assigned based on PCR-RFLP analysis.

Sequence analysis of the entire coding regions of *BMP15* yielded no novel polymorphisms in either wild type individuals or in heterozygous or homozygous carriers of *Fec*X*^G^* or *Fec*X*^B^* from any of the Cambridge, Belclare, Lleyn or HP sheep (Table 4). One point mutation, previously reported in *BMP15* (named B3; [14]) for Belclare ewes was identified in one Lleyn and one Belclare animal. The B3 carriers were heterozygous in both cases and were wild type (Lleyn) and heterozygous *Fec*X*^G^* (Belclare) for the known mutations at this locus.

**Table 4:** Summary of sequence data for complete coding regions of *BMP15* and *GDF9* for animals of various genotypes at these loci and representing four breed groups.

Locus	Genotype	Breed group	Number of animals	SNPs other than those listed under genotype	
				Carriers detected†	No. of homozygous carriers‡
*BMP15*	+/+	Cambridge	4	None (4)	-
		Belclare	4	None (4)	-
		Lleyn	6	None (4), B3 (2)	None
	*Fec*X*^G^*/*Fec*X*^G^*	Cambridge	4	None (4)	-
		Lleyn	5	None (5)	-
	*Fec*X*^G^*/+	Belclare	4	None (3), B3	None
		Lleyn	4	None (4)	-
	*Fec*X*^B^*/*Fec*X*^B^*	Belclare	4	None (4)	-
	*Fec*X*^B^*/+	HP	5	None (5)	-
*GDF9*	+/+	Cambridge	7	None (2), G2, G3 (4), G5 (2), G6 (4), G7 (5)	G2 (1), G3 (1), G7 (1)[haplotypes: G2G7]
		Belclare	7	G2 (3), G3 (7), G4 (3), G5, G6, G7 (3)	G2 (2), G3 (3), G4 (1)[haplotypes: G2G3, G3G4]
		Lleyn	4	None, G3 (3), G5, G6	G3 (1)
		HP	1	G3	None
	*Fec*G*^H^/Fec*G*^H^*	Cambridge	8	None (8)	-
		Belclare	6	None (6)	-
		Lleyn	1	None	-
	*Fec*G*^H^/+*	Lleyn	4	None, G2, G3 (2), G4	None
		HP	1	G6	None

† Number of cases given in parentheses where >1.

‡ Number of cases in parenthesis.

Analysis of the coding regions of *GDF9* yielded six out of seven previously reported point mutations that were classified as without effect on ovarian function, and named as G2, G3, G4, G6 and G7 [14], across the Cambridge, Belclare, Lleyn and HP animals (Table 4). None of these polymorphisms was linked to *Fec*G*^H^* as none of the mutations was present in any of the 15 individuals (eight Cambridge, six Belclare and one Lleyn) that were homozygous for this mutation (Table 4); also there were no homozygous carriers of any of these point mutations among the set of *Fec*G*^H^/*+ individuals (Table 4).

## Discussion

Flocks on the Lleyn peninsula were the source of the Lleyn sheep used in the genesis of the Belclare and it is highly likely that the Lleyn ewes that contributed to the Cambridge came from the same locality, since the breed was not widely known in Britain up to the late 1970s. This, together with the fact that both mutations are segregating in Lleyn flocks in this locality suggests that the Lleyn was the source of these two mutations for both the Cambridge and Belclare breeds. This proposition is consistent with the absence of any difference in the DNA sequence of the relevant coding regions between Belclare, Cambridge and Lleyn carriers. The presence of the *Fec*X*^B^* mutation among the set of HP ewes while it was not found in the Lleyn or in any of the other breeds tested suggests that the High Fertility line was the source of this mutation. However, this conclusion must be qualified by the possibility that the carriers may in fact have had Belclare ancestry. In order to reduce the likelihood of this explanation those animals that could only be described as Suffolk-x were disregarded, both on the basis of the limited information on breed composition but also on the basis of follow-up discussions with the owners and taking into account the geographical location of the flocks concerned (when combined with knowledge that Belclare-cross animals were likely to be local to the region involved). The latter consideration was informed by the pattern of distribution of rams either released from the Teagasc flock in the early stages of on-farm testing of the Belclare breed, or as areas into which Belclare breeders commonly sold rams. Based on these constraints, only two of the five cases involving the *FecX^B^* mutation merited further consideration. Of these, ewe E2170616∶10 is the most compelling case given its location and the information on ancestry provided; the flock involved was very small and the dam of ewe E2170616∶10 had produced quadruplets while the grandmother (described as a Milford ewe) had produced quintuplets (in 1994). No rams with Belclare ancestry were used on this farm and the flock owner was clear that the grandmother, which was purchased, had Scottish Blackface and Leicester ancestry. While there is no authoritative definition of what constitutes a Milford ewe, expert opinion in Donegal indicates that the term is used locally to describe crossbred ewes with Scottish Blackface and lowland-breed ancestry. The possibility that *FecX^B^* may be segregating in the Scottish Blackface breed should be considered, and warrants further investigation.

The background evidence on ewe H2351074∶5 is somewhat less certain; she was by a Texel ram out of a Texel × Cheviot homebred ewe from a small set of Cheviot ewes that her owner maintained. The owner was adamant that he never had a Belclare ram. It is known, however, that one breeder of Belclare sheep regularly sold rams into a contiguous region of Co. Kerry during the 1990s. It seems reasonable to conclude that ewe E2170616∶10 carried a copy of the *Fec*X*^B^* mutation that was not derived from the Belclare breed and it is arguable that the same is true of ewe H2351074∶5. Although genotyping of High Fertility line was not possible as the line was incorporated into the Belclare breed, the evidence from the HP ewes identified for the present study suggests that the *Fec*X*^B^* mutation was present in the set of prolific ewes assembled during the 1960s for the formation of the High Fertility line. This conclusion is supported by the fact that Hanrahan [26] noted the occurrence of ewes in the High Fertility line with ‘abnormalities of the ovaries or uterus’ among a set of ewes that had failed to lamb over three annual joinings. The abnormal ovaries can be equated, in retrospect, with the sterility phenotype found in homozygous carriers of either the *BMP15* or *GDF9* mutations as reported by Hanrahan et al. [14].

While the *Fec*X*^G^* mutation was identified in one HP ewe it is argued that this case should be ignored because of the limited information on breed composition but also because the flock in which it was detected was located very close to the Teagasc centre where the Belclare breed was developed and was also close to the Blindwell test farm where Teagasc evaluated a range breed crosses, including an extensive evaluation of Belclare and Belclare-cross ewes during the 1980s. This conclusion is strengthened by the fact that follow-up discussions with the owner indicated the possibility that a Belclare x Galway type was among the ancestry of the ewe in question.

**Figure 2:**
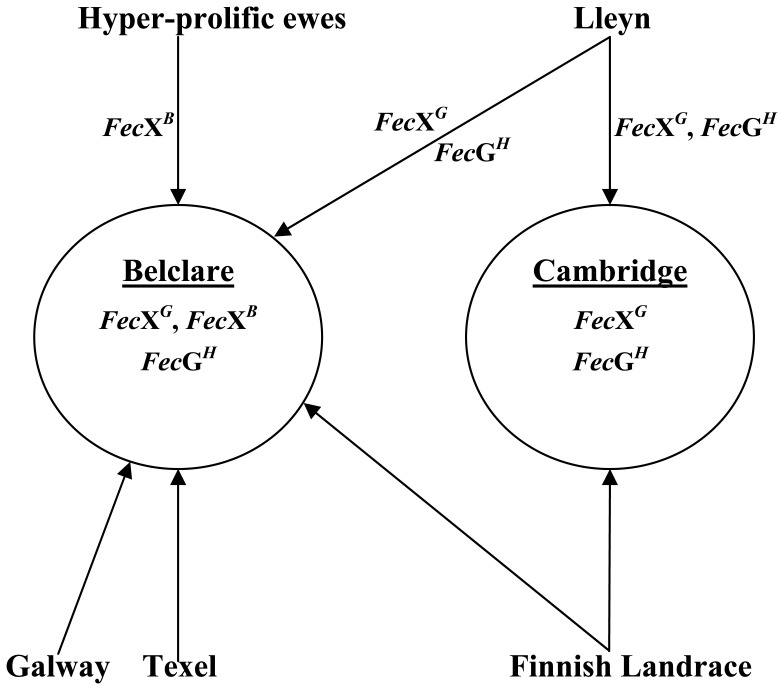
Breeds examined as possible sources of the mutations in *BMP15* (*Fec*X*^G^*, *Fec*X*^B^*) and *GDF9* (*Fec*G*^H^*) in Belclare and Cambridge sheep and sources identified (indicated by mutations associated with arrows). The Hyper-prolific ewes served as a proxy for the High Fertility line used in the development of the Belclare.

The links between the Belclare and Cambridge breeds and the populations studied as possible sources of the mutations in *BMP15* and *GDF9* that are present in these two breeds are summarized in Fig. 2. The conclusions from the present work about the origin of the various mutations are also shown. The mutations in *BMP15* and *GDF9* that are present in the Lleyn can explain the cases of ovarian hypoplasia reported by Vaughan et al. [32] in a flock of Lleyn sheep.

The results from the Lleyn survey confirmed the presence of both *Fec*X*^G^* and *Fec*G*^H^* in the current Lleyn population; the carriers occurred throughout Britain and Ireland. No evidence was found for the presence in the Lleyn of any of the other known mutations with large effect on fertility, from either MassArray® iPLEX genotyping of rams for *Fec*X*^B^*, *Fec*X*^I^*, *Fec*X*^H^*, *Fec*X*^L^ and FecB^B^* or from DNA sequencing of the *BMP15* locus (15 ewes) and the *GDF9* locus (9 ewes) spanning the coding regions encompassing the recently reported mutations *Fec*X*^R^* [17]), *Fec*G*^T^* [18] and *Fec*G*^E^* [19], respectively. It remains unknown whether the unidentified mutations affecting ovarian function in Cambridge [33], Davisdale [34] and Lacaune sheep [35] are present in the Lleyn population.

Since the discovery of mutations with a large effect on prolificacy the role of many these mutations in the prolificacy of other flocks has been examined in numerous studies. However, the only mutations identified (to date) outside the breeds in which they were originally discovered are the *Fec*B*^B^* mutation in the Indian Garole, Indonesian Javanese and the Small-tail Han and the Hu sheep of China [12], [36], and the *Fec*X*^G^* mutation in the Small-tail Han sheep [37]. Given the relatively large number of known mutations at the *BMP15* locus it is seems reasonable to suggest that the *Fec*X*^G^* mutation in the Small-tail Han represents an independent mutation rather than reflecting some common origin with the Lleyn, given the geographic distance/barriers involved and the absence of phylogeographic evidence for any recent connection between British breeds and native Chinese breeds.

The identification of *Fec*X*^B^* and *Fec*G*^H^* in the sample of HP animals from the commercial sheep population in Ireland and the presence of both *Fec*X*^G^* and *Fec*G*^H^* mutations in the Lleyn breed is of relevance to the sheep industry and may warrant a larger study into the frequency of these mutations on farms and the implications for national genetic evaluation programmes.
